# Alcohol Use Disorders and Their Harmful Effects on the Contractility of Skeletal, Cardiac and Smooth Muscles

**DOI:** 10.3389/adar.2021.10011

**Published:** 2021-10-14

**Authors:** Jerusalem Alleyne, Alex M. Dopico

**Affiliations:** Department of Pharmacology, Addiction Science, and Toxicology, College of Medicine, The University of Tennessee Health Science Center, Memphis, TN, United States

**Keywords:** alcohol, alcoholism, ethanol, muscle contractility, myopathy, negative inotropism, vasoconstriction, vasodilation

## Abstract

Alcohol misuse has deleterious effects on personal health, family, societal units, and global economies. Moreover, alcohol misuse usually leads to several diseases and conditions, including alcoholism, which is a chronic condition and a form of addiction. Alcohol misuse, whether as acute intoxication or alcoholism, adversely affects skeletal, cardiac and/or smooth muscle contraction. Ethanol (ethyl alcohol) is the main effector of alcohol-induced dysregulation of muscle contractility, regardless of alcoholic beverage type or the ethanol metabolite (with acetaldehyde being a notable exception). Ethanol, however, is a simple and “promiscuous” ligand that affects many targets to mediate a single biological effect. In this review, we firstly summarize the processes of excitation-contraction coupling and calcium homeostasis which are critical for the regulation of contractility in all muscle types. Secondly, we present the effects of acute and chronic alcohol exposure on the contractility of skeletal, cardiac, and vascular/ nonvascular smooth muscles. Distinctions are made between *in vivo* and *in vitro* experiments, intoxicating vs. sub-intoxicating ethanol levels, and human subjects vs. animal models. The differential effects of alcohol on biological sexes are also examined. Lastly, we show that alcohol-mediated disruption of muscle contractility, involves a wide variety of molecular players, including contractile proteins, their regulatory factors, membrane ion channels and pumps, and several signaling molecules. Clear identification of these molecular players constitutes a first step for a rationale design of pharmacotherapeutics to prevent, ameliorate and/or reverse the negative effects of alcohol on muscle contractility.

## INTRODUCTION

Alcohol (ethyl alcohol; ethanol) has been part of the human diet for approximately 9,000 years and remains one of the most consumed beverages globally (1, 2). While there is still some controversy on the beneficial effects of moderate alcohol consumption, it is established that alcohol misuse has deleterious effects on personal health, family and societal units, and global economies. In 2016 alone, 2.8 million deaths were attributed to alcohol use disorders (AUD) (3). Additionally, the levels of alcohol intake during moderate to heavy drinking (e.g., during binge drinking–see below-), were almost double that of levels considered legal intoxication in most societies (3). A survey from 2019 revealed that 25.8% of Americans over the age of 18 admitted to participating in binge drinking within the previous month (4, 5). Importantly, there are different ranges for drinking levels between men and women, with “a drink” consisting of 14 g of pure alcohol (6). For biological men, moderate drinking is defined as two drinks per day while fifteen or more per week constitutes heavy drinking. For biological women, however, one drink per day defines moderate drinking while eight or more per week reflect heavy drinking (7). Thus, binge drinking reflects 5 drinks for men or 4 drinks for women over a 2 h period, which corresponds to a blood alcohol concentration of 0.08% (8).

Most studies on the negative effects of alcohol abuse on the body involve biological males. However, there is compelling evidence that some of the adverse effects and deleterious consequences of alcohol abuse (e.g., development of cirrhosis and hepatitis at an earlier age, aberrant anabolic signaling pathways and decreased protein synthesis), are more severe in biological females (9-11). These differences are largely a consequence of sex differences in the bioavailability of ethanol, which is determined by absorption, distribution, metabolism and elimination (ADME) processes. Indeed, it has been documented that all ADME processes contribute to determine the increased susceptibility of women to alcohol-induced myopathies and diseases (9, 12, 13). Before ethanol enters the bloodstream, it is firstly metabolized in the gastrointestinal system by gastric alcohol dehydrogenase (ADH). Despite exhibiting higher liver oxidation and overall elimination of ethanol when compared to men (9), women exhibit lower gastric ADH activity. Overall, the extent of alcohol first-pass metabolism is greater in men than in women what results in higher blood alcohol levels of ethanol for a protracted length of time in females (12, 14).

In both sexes however, alcohol abuse (i.e., increased alcohol intake as result of higher frequency of drinking and/or increased amount of alcohol per session) usually leads to many diseases, including establishment of the chronic condition of alcoholism, alcoholic liver disease, oral cavity and esophageal cancers (15-18). In particular, alcohol abuse, whether acute or chronic, adversely affects skeletal, cardiac and/or smooth muscle contraction and eventually leads to various myopathies (15, 18-20). In skeletal muscle, acute alcohol intoxication causes symptoms such as muscle weakness and swelling while chronic abuse causes intensifying muscle pain, chronic inflammation and loss of muscle mass which, at the skeletal fiber level, have been related to micronutrient deficiency and mitochondrial dysfunction (18). In the cardiovascular system, moderate to heavy alcohol consumption can lead to hypertension, arrhythmias, stroke and even heart failure, with dysfunction of the contractile machinery contributing to these outcomes (7, 15-18). Likewise, the contractile function of both vascular and non-vascular smooth muscle is disrupted by alcohol abuse (21-28). With some notable exceptions to be discussed under subheadings below, it is reasonable to advance that alcohol actions on the contractility of all muscle types (skeletal, cardiac and smooth muscle) are primarily carried out by ethanol itself, irrespective of alcoholic beverage type and ethanol metabolites (15,16). Being that ethanol is a very simple molecule, it is considered a “promiscuous ligand”, i.e., able to interact with multiple molecular targets and simultaneously interfere with varied cell signaling mechanisms to evoke a define cellular effect (29). Ethanol-myocyte interactions are not exception.

Under the next subheading, we introduce the reader to a brief overview of skeletal, cardiac and smooth (vascular and nonvascular) muscle contraction, with a focus on participating molecules and mechanisms at the myocyte level. Then, we describe the effects of alcohol on these elements which in turn impact muscle contraction, and the eventual development of alcoholic myopathy in the different types of muscular tissue.

## MUSCLE TYPES AND THEIR MECHANISMS FOR CONTRACTION

In developed mammals there are three major types of muscle: skeletal, cardiac and smooth muscles. Succinctly, skeletal muscles move body parts (mainly bones to which they are attached *via* tendons) resulting in motion. The cardiac muscle or myocardium pumps blood through and from the heart to the periphery, and smooth muscles constrict or relax to keep a necessary state of partial muscle contraction (myogenic tone) for proper organ function, e.g., for proper flow of blood from vessels to organs. All three types of muscle, however, contain contractile proteins such as actin, myosin, troponin and tropomyosin which generate physical force, resulting in muscle contraction (30-33). Skeletal and cardiac muscles are called striated muscles because actin and myosin are arranged in complex arrays called sarcomeres. In contrast, the actin and myosin in smooth muscle are not arranged in sarcomeres, and the total amount of contractile protein is 25% less than what is found in striated muscles (31, 32). With respect to size, striated muscle myocytes are much larger in size than their vascular smooth muscles counterparts. In general, smooth muscle cells retain some proliferative capability unlike striated muscles which are essentially post-mitotic (32).

In all muscle cell types, the second messenger Ca^2+^ is involved in many essential processes such as gene regulation, apoptosis, autophagy and cell survival (34, 35). In particular, the increased availability of Ca^2+^ into the cytosol, from extracellular Ca^2+^ influx and/or Ca^2+^ release from intracellular stores, is essential to generate contraction in both striated and smooth muscles. Therefore, cytosolic Ca^2+^ levels are tightly buffered, which largely results from this ion being sequestered in intracellular organelles that include the endo/sarcoplasmic reticulum (ER/SR), mitochondria and lysosomes (34-40).

Inositol 1,4,5-trisphosphate receptors (IP_3_R) and ryanodine receptors (RyR) are the main intracellular Ca^2+^ release channels embedded in the ER/SER membranes (34, 36-38). Both receptor families evolved from a common ancestor and share ~40% homology (34, 35). Moreover, both receptor types form large homo-tetrameric complexes when inserted into membranes. However, their functions and downstream consequences are highly specialized and distinct even within a given cell type. Both IP_3_Rs and RyRs have three isoforms; RyR1, RyR2 and RyR3 are the skeletal-, cardiac- and brain-“specific” isoforms of RyR (it should be noted, however, that RyR3 have a more widespread expression, including vascular smooth muscle; see below). Their corresponding IP_3_R1, IP_3_R2 and IP_3_R3 isoforms however, are expressed in many different tissues; IP_3_R1 is the most abundant in most cell types while IP_3_R2 is most highly expressed in cardiac tissue (34, 35).

RyR-mediated Ca^2+^ release from SR stores is the central mechanism that leads to contraction of striated fibers while IP_3_Rs play a more substantial role in the contraction of smooth muscle (36, 38-40). The mechanisms involved in RyR1 and RyR2 function/regulation in skeletal, cardiac and smooth muscle have been widely studied; however, less is known about RyR3 (41-43). In turn, the main targets of IP_3_R-mediated Ca^2+^ release are the mitochondria, which in turn regulates cell metabolism, lysosome activity and autophagy. IP_3_R is modulated by a canonical IP_3_R signaling pathway along with other regulatory proteins (34-37).

### E-C Coupling in Striated Muscle and Smooth Muscle Cells

**FIGURE 1 ∣:**
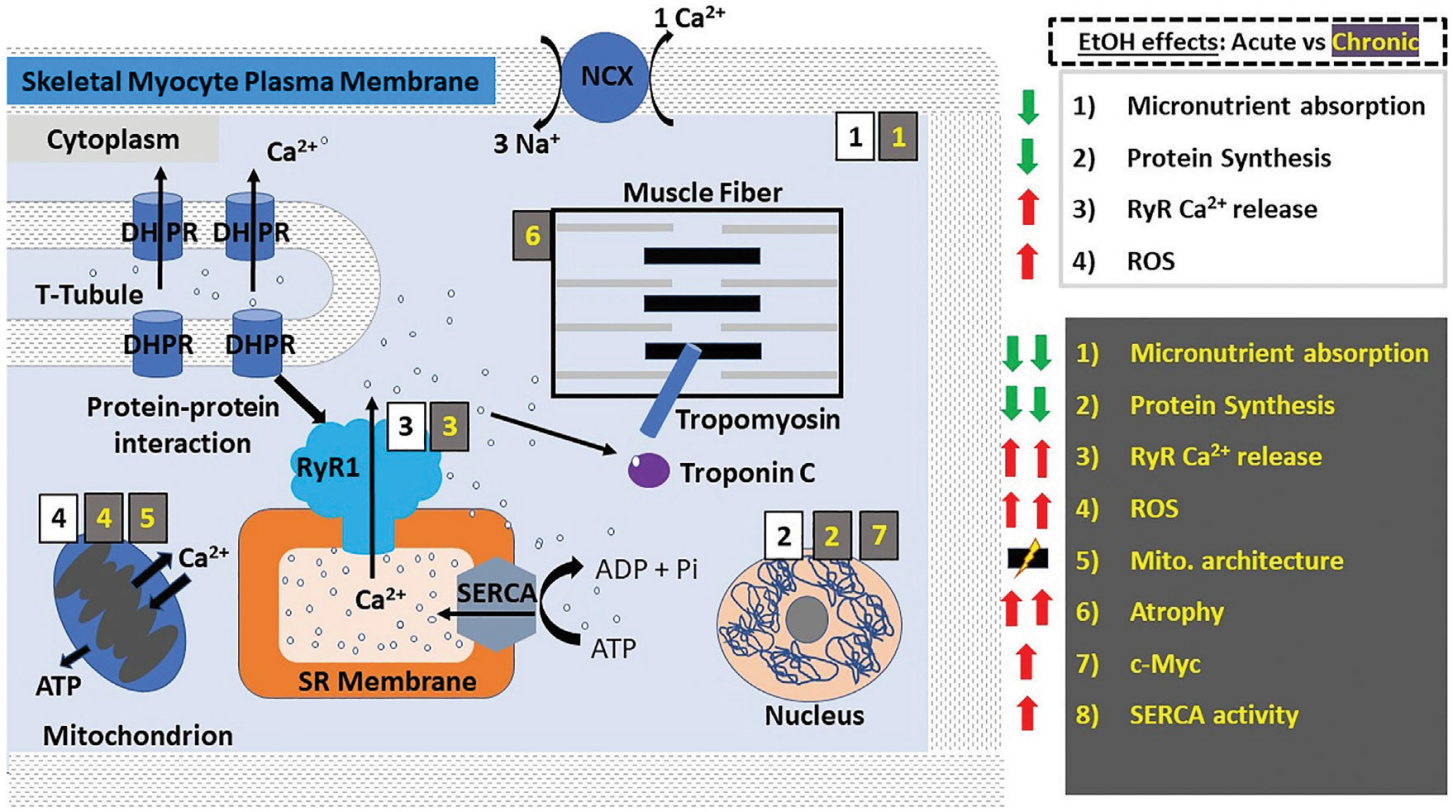
Alcohol actions on skeletal muscle myocyte contractility. In skeletal muscle myocytes, E-C coupling is mediated by the physical interaction between DHPRs on the t-tubules and RyR1 on the SR membrane; membrane depolarization activates DHPRs leading to their mechanical coupling with and eventual activation of RyR1, which in turn releases Ca^2+^ from SR stores. The resulting influx of Ca^2+^ into the cytoplasm causes the activation of troponin C, which activates tropomyosin leading to a change in its conformation and allowing myosin and actin to associate resulting in muscle contraction by way of a “power stroke”. Here and in all other figures, the main actions of acute and chronic ethanol consumption are shown with black numbers on white background and with yellow numbers on gray background, respectively. Acute ethanol consumption/administration causes decreased micronutrient absorption and protein synthesis while increasing RyR1-mediated Ca^2+^ release and the production of ROS. Chronic ethanol consumption/administration exacerbates the aforementioned effects and leads to increased SERCA re-uptake of Ca^2+^ into the SR, disruption of mitochondrial architecture and the predisposition to and development of skeletal muscle atrophy, which is thought to involve ethanol-induced upregulation of the proto-oncogene c-Myc. Abbreviations: DHPRs, dihydropyridine receptors; NCX, Na^+^/Ca^2+^-exchanger; ROS, reactive oxygen species; RyR, ryanodine receptors; SERCA, SR Ca^2+^ transport ATP-ase; SR, sarcoplasmic reticulum; t-tubules, transverse tubules.

**FIGURE 2 ∣:**
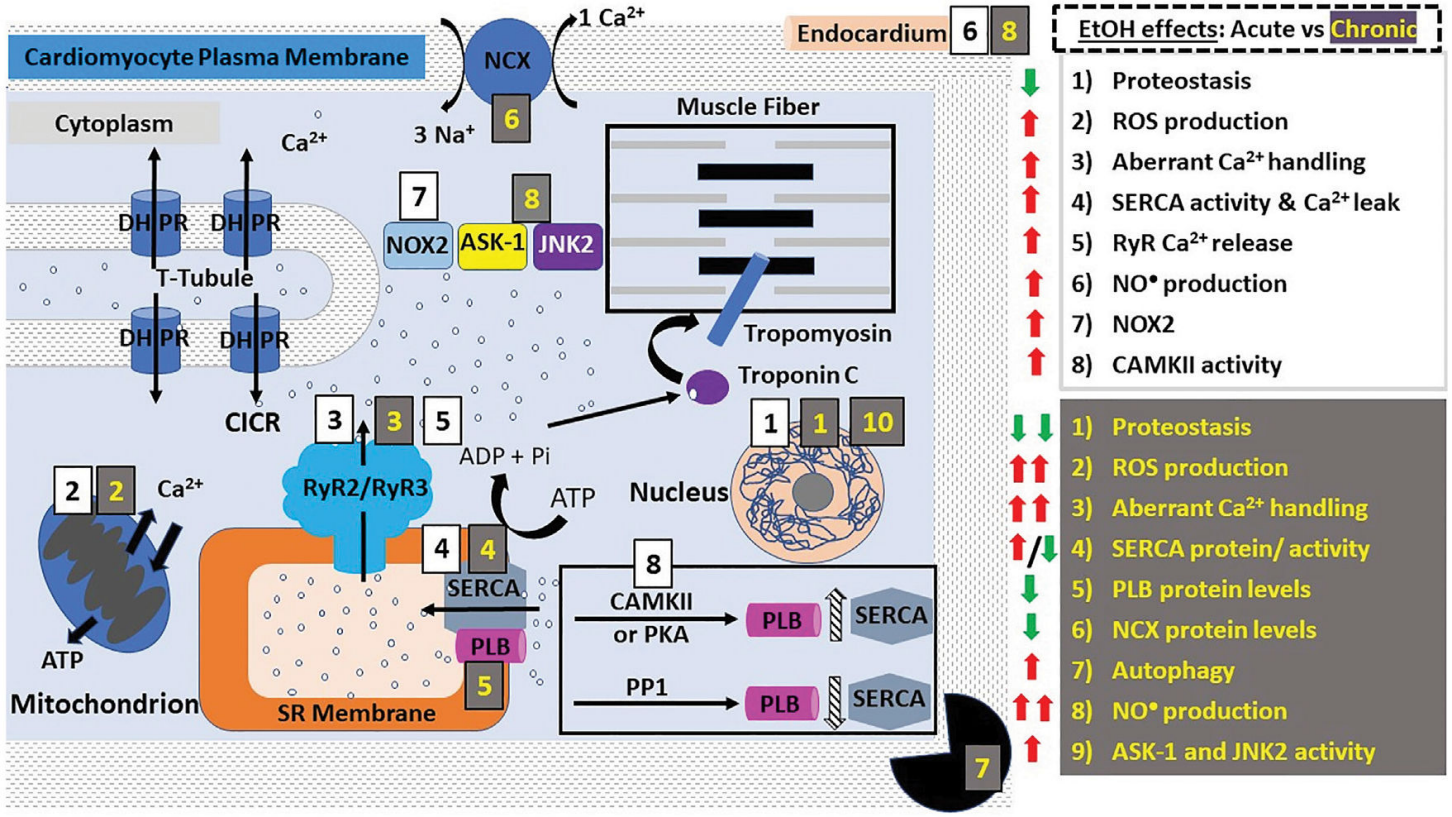
Alcohol action on cardiomyocyte contractility. E-C coupling in cardiomyocytes occurs *via* calcium-induced calcium release (CICR). DHPRs are activated by depolarization of the cardiac myocyte membrane (sarcolemma) causing them to release a small amount of Ca^2+^ into the cytoplasm. This Ca^2+^ then activates RyR2, leading to Ca^2+^ influx from SR stores and an exponential increase in the intracellular Ca^2+^ concentration. Ca^2+^ then binds and activates troponin C which activates tropomyosin, allowing the physical interaction between myosin and actin. The points of interaction between these two contractile proteins are called “cross bridges” and allow myosin heads to slide across actin filaments, resulting in a “power stroke” and myocyte contraction. Both acute and chronic alcohol consumption lead to negative inotropic effects (diminished contractility). The effects of acute ethanol consumption/administration include: decreased proteostasis (decreased protein synthesis and altered function etc.), increased ROS production and oxidative stress, decreased Ca^2+^ handling (see main text), increased SERCA activity and increased NO^•^ production. NOX2 signaling and CAMKII activity were shown to be involved in ethanol-induced increase in ROS production. Chronic ethanol consumption/administration exacerbates these effects. In addition, other effects are observed such as increased autophagy and significantly decreased protein levels of SERCA, NCX, CYP-2E1, iNOS and PLB. The significant increase in ROS production and oxidative stress was shown to be linked to the ethanol-mediated upregulation of JNK2 and ASK-1 signaling pathways. These alcohol-induced negative inotropic events serve to reduce cardiac contractility and increase susceptibility to the development of various cardiomyopathies such as AF. Abbreviations: ASK-1, Apoptosis signal-regulating kinase 1; CAMKII, Ca^2+^ calmodulin-dependent protein kinase II; DHPRs, dihydropyridine receptors; JNK2, c-Jun NH (2)-terminal kinase; NCX, Na^+^/Ca^2+^-exchanger; PKA, protein kinase A; PLB, phospholamban; PP1, protein phosphatase 1; ROS, reactive oxygen species; RyR, ryanodine receptors; SERCA, SR Ca^2+^ transport ATP-ase; SR, sarcoplasmic reticulum; t-tubules, transverse tubules.

Excitation-contraction (E-C) coupling describes the events starting from the generation of an action potential (AP) to muscle contraction, and is a mechanism utilized by all three muscle types. In striated muscles (skeletal and cardiac), depolarization *via* AP reaches the so-called t-tubules (i.e., transverse tubules), which are specialized regions of the sarcolemma that protrude deep into the cell. Herein, the depolarizing wave activates L-type voltage-gated (Cav1.1/Cav1.2) Ca^2+^ channels (also known as dihydropyridine receptors: DHPR), which are abundant in the t-tubules (44, 45). However, E-C coupling in heart and skeletal muscle is not identical. In skeletal myocytes, depolarization-activated DHPR mechanically engage with sarcoplasmic RyR1 through protein-protein interactions, leading to RyR1 activation and eventual release of sarcoplasmic Ca^2+^ into the cytoplasm and therefore skeletal fiber contraction (Figure 1). In addition, the mitochondria are packed tightly around the contractile proteins and are also connected to the SR membrane. These interactions are critical for E-C coupling and Ca^2+^ homeostasis to occur because mitochondria supply the critical energy mediator ATP and also collect some of the Ca^2+^ released by RyR1 (46, 47). In cardiac fibers, there is no evidence of direct physical coupling between DHPR and RyR. The depolarization-dependent activation of DHPR, however, leads to Ca^2+^ influx, with this Ca^2+^ thus being bound by sarcoplasmic RyR2, which then releases Ca^2+^ from the SR (Figure 2). This process has been termed Ca^2+^-induced Ca^2+^ release (CICR). While E-C coupling is different in cardiac and skeletal myocytes, RyR activation in both striated muscles results in the release of Ca^2+^ from SR stores and an ~10x increase in the cytoplasmic concentration of Ca^2+^ (34, 45).

**FIGURE 3 ∣:**
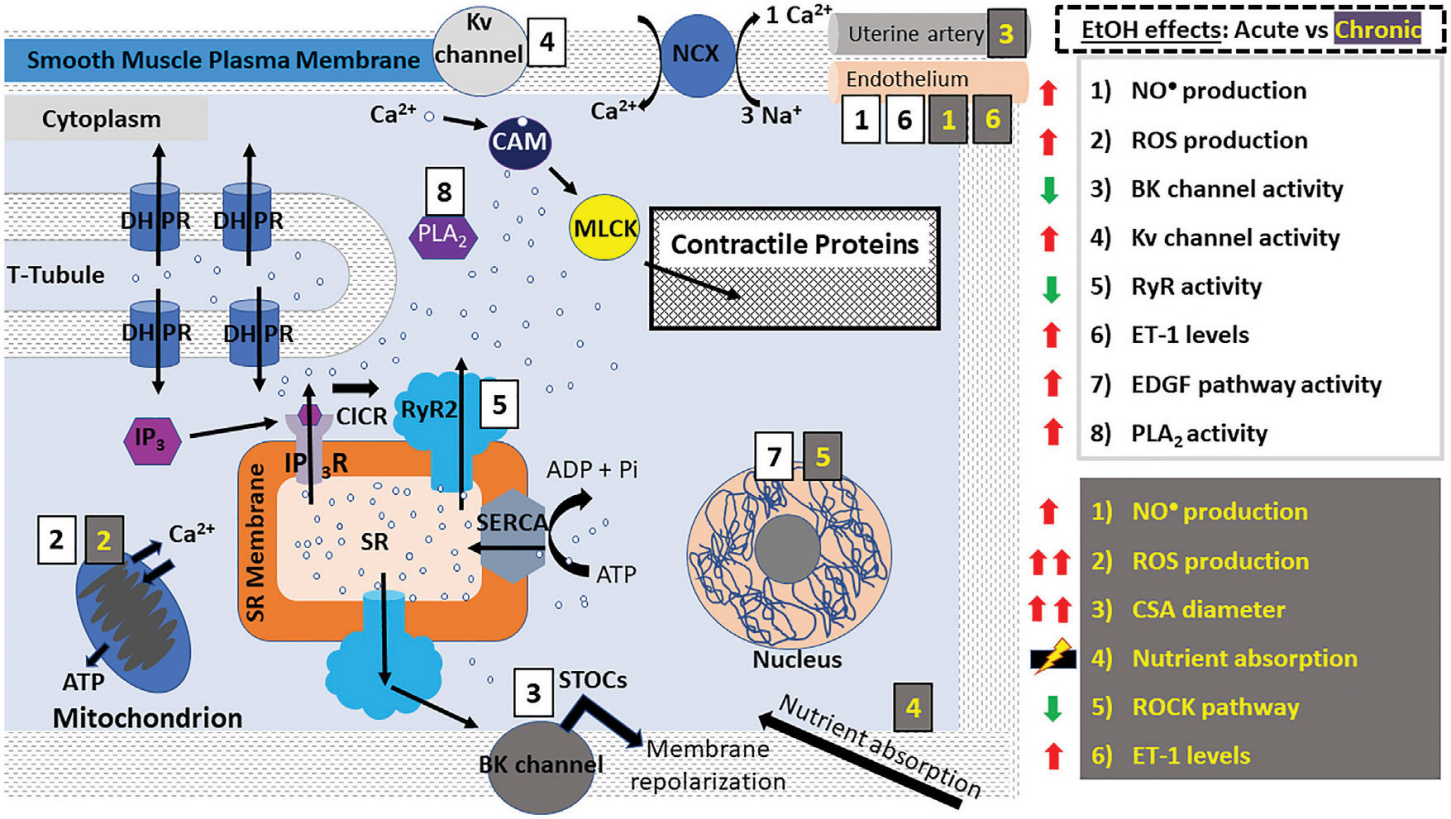
Alcohol action on smooth muscle myocyte contractility. E-C coupling in smooth muscle myocytes occurs *via* calcium-induced calcium release (CICR) mechanisms as seen in cardiomyocytes. However, Ca^2+^-release leading to contraction occurs signficantly *via* IP_3_Rs. DHPRs are activated by depolarization of the smooth muscle myocyte membrane causing RyR2 and IP_3_Rs to release a small amount of Ca^2+^ into the cytoplasm. Unlike striated myocytes, troponin and tropomyosin are not involved in the coupling of myosin and actin; instead, Ca^2+^ ions bind to CAM which phosphorylates MLCK leading to its association with actin, resulting in a “power stroke”. In vascular smooth muscle, RyR2 (and possibly RyR3 as well)-induced release of Ca^2+^ generates the so-called “sparks” which activate BK channels, leading to membrane repolarization and vasodilation. In smooth muscle myocytes, alcohol has been reported to cause both contraction and relaxation according to the type of muscle (vascular/ non-vascular), concentration of alcohol used and other conditions. The effects of acute ethanol consumption/administration include: increased ROS and NO^•^ production (aorta, coronary, cerebral and mesenteric arteries), decreased BK channel activity (aorta, cerebral arteries), increased Kv channel activity (coronary arteries), decreased RyR activity (cerebral arteries), increased EDGF activity (mesenteric arteries) and increased PLA2 activity (bladder). Many of these events are exacerbated after chronic ethanol consumption/administration which additionally causes increased uterine artery diameter, decreased ROCK pathway activity (lungs), increased ET-1 levels (carotid arteries, vas deferens) and dysregulation of nutrient and water absorption in the gut. Abbreviations: BK channels, big K^+^ channels; CSA, cross sectional area; DHPRs, dihydropyridine receptors; EDGF, endothelium-dependent hyperpolarizing factor; ET-1, endothelin 1; IP_3_R, inositol trisphosphate receptor; NCX, Na^+^- Ca^2+^-exchanger; PLA2, phospholipase A2; PLB, phospholamban; ROS, reactive oxygen species; ROCK, Rho-associated protein kinase; RyR, ryanodine receptors; SERCA, SR Ca^2+^ transport ATP-ase; SR, sarcoplasmic reticulum; STOCs, Spontaneous Transient Outward Currents; t-tubules, transverse tubules.

In smooth muscle, both IP_3_Rs and RyRs participate in Ca^2+^-release and muscle contraction, *via* mechanisms comparable to the CICR utilized by cardiomyocytes (Figure 3) (34-40, 48-50). In vascular smooth muscle, however, the close vicinity between sarcoplasmic RyR2 (and likely RyR3 as well) and Ca^2+^/voltage-gated K^+^ channels of big conductance (BK channels) located in the sarcolemma leads to BK channel-mediated Spontaneous Transient Outward Currents (STOCs), which oppose depolarization, blunt Ca^2+^-influx and thus, oppose smooth muscle contraction while enabling myocyte *relaxation* and vasodilation. A RyR-generated, local Ca^2+^ transient that activates a STOC is termed a “Ca^2+^ spark” (51-54). In addition to the IP_3_R- and RyR-mediated Ca^2+^ release mechanisms, all muscle types undergo a so-called “Ca^2+^ leak” from ER/SR Ca^2+^ stores, which is a process critical to prevent Ca^2+^ overload in the ER/SR (55-57).

In short, the release of Ca^2+^ into the cytoplasm of skeletal, cardiac and smooth muscle myocytes by RyRs and IP_3_Rs, is coupled to the activation of contractile proteins and muscle contraction as outlined below (30, 32, 33).

### Contractile Proteins and Generation of the “Power Stroke”

The increased Ca^2+^ availability resulting from mechanisms succinctly described in the previous section leads to binding of these ions by contractile proteins present in all muscle types. In striated muscle, actin, myosin, troponin and tropomyosin are the main effectors of muscle contraction (30, 32, 33). During the resting (relaxed) state, tropomyosin physically blocks any interaction between actin and myosin. The influx of Ca^2+^ into the cytoplasm activates troponin by inducing a conformational change in its structure (30, 32, 33). This change in troponin leads to its interaction with tropomyosin, which ultimately removes the latter from actin filaments. Actin then attaches to myosin leading to the creation of cross bridges, which are the point at which actin begins to slide across the myosin filaments in an ATP-dependent manner. This motion, called a “power stroke”, shortens the muscle cell resulting in contraction (30, 32, 33).

Unlike striated muscles, smooth muscle cells do not contain troponin. Instead, phosphorylation of myosin regulatory light chains (RLC) governs smooth muscle contraction (30, 33, 58). Ca^2+^ activates calmodulin (CaM), which in turn phosphorylates myosin light-chain kinase (MLCK). MLCK then phosphorylates myosin RLC leading to the formation of cross bridges with actin, thus resulting in muscle contraction. While not predominant, this mechanism also operates in cardiac myocytes (30, 58).

To prevent a permanent state of muscle contraction, the intracellular Ca^2+^ concentration is diminished immediately after cell contraction (59-61). This results in the reversion of troponin to its original conformation, thereby allowing tropomyosin to bind actin and prevent its association with myosin, with the myocyte returning to its resting state. Thus, Ca^2+^ removal from the myocyte is tightly regulated and involves diverse ion channels and pumps (30, 59-61). The most critical element in replenishing ER/SR stores with Ca^2+^ is the activity of the SR Ca^2+^ transport ATP-ase (SERCA); which actively pumps Ca^2+^ from the cytoplasm into the intracellular SR stores. Ca^2+^ uptake by SERCA is tightly regulated by phospholamban (PLB) in the heart and smooth muscle (59-62). PLB interacts with SERCA to decrease Ca^2+^ uptake, however this is reversed upon PKA-mediated phosphorylation of PLB, and the resumption of SERCA-mediated Ca^2+^ uptake (59-61). Apart from SERCA, cytoplasmic Ca^2+^ is also transported to the extracellular space by the sarcolemma Ca^2+^-ATPase pump and the Na^+^-Ca^2+^-exchanger (NCX) pump (57).

Collectively, the summary above highlights the wide variety of molecular entities involved in muscle contraction (ion channels, ionic pumps, contractile proteins and/or their regulatory proteins). Therefore, they are all putative molecular targets of ethanol which mediate, or at least contribute to, alcohol-induced disruption of muscle contraction in the three different muscle types. Indeed, many ion channels have been identified as potential molecular targets of ethanol, in particular those contributing to intracellular Ca^2+^ homeostasis (23, 42, 63-65). The ffects of ethanol on the mechanisms directly involved in E-C coupling, intracellular Ca^2+^ homeostasis and regulation of contractile proteins in muscle cells are the main foci of the following subsections.

### Alcohol and Skeletal Muscle

The physical signs of alcohol abuse include impaired motion, skeletal muscle atrophy (loss of skeletal muscle mass) and muscle weakness (66). Remarkably, alcohol-induced skeletal myopathies significantly outnumber inherited myopathies (18,66,67). The pathological consequences of acute alcohol abuse include muscle tissue breakdown with the release of muscle content into the blood and elevated levels of creatinine kinase and myoglobin, decreased micronutrient absorption and reduced protein synthesis (18, 66-68). These abnormalities are also observed after chronic alcohol abuse, which additionally evokes further muscle dysfunction, impaired muscle regeneration, increased risk for muscle injuries, muscle weakness, pain, and localized atrophy (11, 18, 66-68).

Despite this plethora of signs and symptoms, the most common effect of alcohol-related skeletal myopathies is skeletal muscle atrophy, which is present in over 50% of chronic alcohol users (69). The main driver of alcohol-induced skeletal muscle atrophy is thought to be the decline in protein synthesis, though the exact mechanisms of these ethanol actions in skeletal muscle are unknown (66-69). In particular, the levels of proteins that participate in muscle contraction and elasticity, such as nebulin, titin and myosin heavy chain protein, have been shown to be significantly decreased (11, 68). Moreover, ethanol-mediated upregulation of reactive oxygen species (ROS) has also been identified as a major player in the damage of proteins, inhibition of protein synthesis and upregulation of proteolysis in skeletal muscle (66, 70, 71). Crowell et al. (2019) (68) compared the effect of chronic and acute alcohol consumption on murine skeletal muscle mass and function. In this experimental model, acute alcohol administration (a single dose) did not impair skeletal muscle function, in contrast to what has been seen in humans (72). In turn, chronic ethanol administration to mice significantly decreased muscle contractility and shortened the time taken for muscles to become fatigued (68). In humans, alcohol-induced atrophy, though reversible upon abstinence, can become permanent without the cessation of drinking (18, 66, 68).

In contrast to the wealth of knowledge about the different subcellular mechanisms by which alcohol affects cardiac and smooth muscles, such information is scarce for skeletal muscle. Regardless of the molecular underpinnings, it is established that ethanol affects not only skeletal myofibrillar function but also their structural organization (47, 66, 69). Collectively, gene expression of growth and fibrotic factors is decreased, autophagy and ubiquitin-proteasome pathways are dysregulated, inflammation and oxidative stress are induced, and mitochondrial function is perturbed (66, 69). Indeed, mitochondria have been particularly identified as targets for ethanol actions in the muscle (47). In *C. elegans*, which has muscle structures comparable to mammalian striated muscles (69), ethanol exposure disturbed mitochondrial architecture, upregulated stress response genes and induced oxidative and ER stress. Upregulation of the mitochondrial unfolded protein response system (UPR^mt^), however, alleviated these deleterious alcohol-induced effects, thereby improving mitochondrial function and skeletal muscle contractility (69).

#### Alcohol and E-C Coupling in Skeletal Muscle

Regarding the different mechanisms that control Ca^2+^ homeostasis in skeletal fibers, Ohlendieck et al., (2003) (73), revealed that chronic alcohol administration increased SERCA1 and Ca^2+^-ATPase protein levels. In turn, Cofan et al. (1995) (74) showed a depletion of intracellular Ca^2+^ when muscle was exposed to ethanol (20–200 mM). Studies on RyR1 modulation by ethanol, however, are scarce. More importantly, the available studies differ in methodology, experimental conditions and results, making it difficult to reach a definitive conclusion on ethanol-mediated regulation of RyR1 function. For example, acute administration of ethanol (2–20 mM), increased Ca^2+^release from heavy SR fractions isolated from rabbit skeletal muscle (41). Moreover, preliminary data from our laboratory showed that ethanol (50–100 mM) was able to increase the steady-state activity of recombinant RyR1 reconstituted into artificial phospholipid bilayers (75). These data indicate that RyR1 is a pharmacological target of ethanol at concentrations reached in blood during alcohol intoxication. The contribution of this alcohol action to skeletal fiber contractility, however, remains to be determined. In contrast to the activatory effects of alcohol on RyR1 considered above, pre-treatment of bullfrog SR vesicles with 2.2–217 mM ethanol had no effect by itself, yet Ca^2+^ release increased significantly in the presence of both 2.2 mM ethanol and caffeine. Moreover, Cofan et al. (1995) (74) showed that acute exposure to ethanol (20–200 mM) depleted the intracellular Ca^2+^ concentration of resting cultured rat myocytes while chronic exposure failed to do so, indicative of “ethanol tolerance.”

#### Influence of Sex on Alcohol-Induced Skeletal Myopathies

Studies on the alcohol-related skeletal muscle atrophy are predominantly focused on males. However, the deleterious outcomes of ethanol consumption on skeletal muscle function in women are more severe, despite ingesting lower concentrations of ethanol (7, 9-11, 68). It was shown that women at early stages of chronic alcohol abuse possessed decreased levels of titin and nebulin proteins and displayed diminished cross-sectional area of muscle fibers when compared to their male counterparts (11). This sex-sensitive mechanism of skeletal muscle atrophy has been investigated further; there is some evidence of a connection between the proto-oncogene cMyc and skeletal muscle atrophy (76). Indeed, chronic ethanol administration resulted in the upregulation of c-myc expression. This was proposed to be a downstream effect of ethanol-induced corticosteroid expression, which impairs catabolism in skeletal muscle (76). However further investigation needs to be done to confirm these findings and to establish the subcellular mechanisms by which ethanol disrupts skeletal muscle contraction.

### Alcohol and Cardiac Muscle

There is much debate among researchers about the role of alcohol on the cardiovascular system. However, the general consensus is that both chronic and acute consumption of large concentrations of alcohol have deleterious effects on cardiac function, contractility in particular, and increase the risk for developing cardiac conditions such as atrial fibrillation (AF), myocardial infarction, and chronic heart failure (7, 15, 19, 57, 77-79). In contrast, the consumption of low quantities of alcohol is generally believed to offer some protection against cardiovascular disease (78). These viewpoints are discussed below.

#### Acute vs Chronic Alcohol Consumption

Episodic consumption of large quantities of alcohol such as during “binge-drinking” can cause the onset of cardiac arrhythmias, the most common of which is atrial fibrillation (AF) (20, 57, 78, 79). In fact, it has been widely observed that otherwise healthy individuals often developed AF or other arrhythmias after indulging in binge drinking during vacations, holidays or weekends. This observation led to the coinage of the terms “Holiday Heart Syndrome” (HHS) or “Party Heart Syndrome” (20, 78, 79). HHS symptoms include chest pain, fainting and shortness of breath, though some affected individuals may be asymptomatic (78).

The overarching hypothesis that chronic consumption of low to moderate levels of alcohol may play a cardioprotective role, however, has been often termed the “French Paradox” (7, 15, 78, 80-84). This term was derived from the observation that, compared to their counterparts in other developed societies, equivalent French populations presented lower mortality rates, despite the presence of risk factors for developing cardiovascular disease (elevated cholesterol, diabetes, hypertension, etc.,). The French, however, are known for regular (daily) consumption of low amounts of ethanol, usually in the form of red wine (rather than episodic drinking of beer) (7, 78, 83). While the relationship between alcohol intake and risk of cardiovascular events does follow a “J” shape, suggesting cardiovascular protection at low ethanol concentrations (7), a major contention is centered around the exact components of alcohol involved in its cardioprotective effects. Some studies have indicated that ethanol itself is the critical component in providing cardioprotection (80, 84) while others point to the importance of anti-oxidant compounds, more abundant in red wines, such as resveratrol and polyphenols (81, 82). However, the methodology used in collecting these epidemiological data, such as insufficient randomization of the studies, has been criticized (85). Furthermore, recent studies have shown that even low doses of alcohol can increase the risk for developing AF (86).

#### Molecular Mechanisms Behind Alcohol-Induced Cardiac Dysfunction

Several changes at cellular and subcellular levels occur in response to acute and chronic alcohol consumption. At the cellular level, alcohol-induced cardiac dysfunction presents as impaired proteostasis (i.e., altered protein homeostasis), diminished intracellular Ca^2+^ handling and signaling, increased oxidative stress and increased apoptosis, all of which contributing to reduced cardiac contractility (87-89). However, the overall impairment of cardiac function, usually referred to as alcohol-induced cardiomyopathy, involves not only cardiac muscle components but also endothelial, neural and circulating factors (15, 16, 77).

Nevertheless, alcohol is indeed able to exert negative inotropism on cardiac muscle, independently of endothelial, neural, metabolic or circulating factors (16, 56, 87, 89, 90). Some of the biochemical players involved in mediating ethanol-induced negative ionotropic events in cardiac muscle include ROS (H_2_O_2_, O_2_^−^), reactive nitrogen species (nitric oxide: NO^•^), ion channels and associated proteins, such as SERCA, RyR2 and phospholamban (PLB), and acetaldehyde itself (88, 91-96). These molecular players all serve to disrupt Ca^2+^ signaling, which in turn disrupts E-C coupling and thus leads to reduced cardiac contractility (10, 56, 57). The molecular mechanisms used by these entities to mediate ethanol-induced disruption of E-C coupling are described below.

#### ROS, Reactive Nitrogen Species, Ion Channels and the Perturbation of E-C Coupling in the Myocardium

The oxidation of ethanol is carried out by three main enzymes: alcohol dehydrogenase, catalase, and CYP-2E1 (91-95). Alcohol dehydrogenase (ADH) and aldehyde dehydrogenase 2 family member (ALDH_2_) both oxidize alcohols to aldehydes and ketones (91, 95), catalase breaks down H_2_O_2_ to water and oxygen (96) and CYP-2E1 converts ethanol to acetaldehyde (93,95). These enzymes have been linked to the negative inotropic effects of ethanol *via* the production of ROS, which are critical biochemical players in the disruption of skeletal (13, 18, 66, 70, 71), cardiac (56, 88, 94, 96) and smooth muscle function (24, 97, 98).

Mitochondria play an important role in Ca^2+^ sequestration and EC-coupling and are the main source of ROS production (99). Ethanol has been shown to target the mitochondria, where it disturbs the structure and function of the mitochondrial membrane and its overall function as an organelle. One of the critical mitochondrial enzymes involved in ethanol metabolism is ALDH_2_, which is highly expressed in cardiac myocytes and serves to metabolize acetaldehyde (99, 100). The downstream consequences of ethanol-induced mitochondrial dysfunction are increased apoptosis and necrosis (99) thus leading to a decrease in the contractile tissue mass.

The acute exposure of ALDH_2_-knockout mice to ethanol led to significantly increased acetaldehyde production, impaired mitochondrial function, and decreased myocyte contractility when compared to wild type mice (101). In the case of chronic ethanol administration, ALDH_2_ transgenic mice exhibited improved Ca^2+^ handling and homeostasis, increased cell shortening, and decreased apoptosis, with apoptosis signal-regulating kinase 1 (ASK-1) and CREB activity also being implicated in this phenotype (102). Likewise, Brandt et al. (2016) (103) showed that acetaldehyde upregulated NADPH oxidase-2 (NOX2), which has been linked to the onset of heart failure *via* augmentation of ROS production. However, in stark contrast to the preceding findings, low levels of acetaldehyde were found to play a cardioprotective function *via* a mechanism involving ALDH_2_ (100).

Catalase is expressed in the myocardium, albeit in lower quantities in comparison to other organs. Still, it serves to metabolize the harmful and unstable H_2_O_2_ (96). Using ventricular myocytes from transgenic mice, Zhang et al., (2003) (96) showed that catalase overexpression diminished the negative inotropic events induced by acute ethanol administration. Moreover, RyR expression was upregulated and myocardial E-C coupling was improved owing to enhanced Ca^2+^ handling. These authors also found that protein expression of the Na^+^/Ca^2+^ exchanger (NCX) which removes intracellular Ca^2+^, was upregulated. There was no change however, in SERCA, PLB or DHPR expression/activity. Furthermore, AKT signaling was also increased, revealing a possible cardioprotective role of this pathway (104).

Following acute ethanol exposure, transgenic mice expressing the ADH gene, experienced enhanced inotropic events, such as aberrant Ca^2+^ handling and decreased cell shortening, as compared to wt FVB mice (91, 105). These results indicated the involvement of increased acetaldehyde levels in the onset of negative inotropic events and the dysregulation of cardiac contractility. Additionally, it was shown that simultaneous inhibition of catalase and ADH ablated the negative inotropic effects induced by acute ethanol consumption in female rats, further underscoring the involvement and importance of these enzymes to the deleterious effects of ethanol and acetaldehyde on cardiac muscle (95).

NO^•^ is a gaseous signaling molecule produced endogenously from the breakdown of L-arginine by NO^•^ synthetases (NOS). There are three NOS isoforms: endothelial (eNOS), neuronal (nNOS) and inducible (iNOS), which regulate the activity of many proteins involved in cardiac Ca^2+^ homeostasis and E-C coupling, such as L-type Ca^2+^ channels (DHPR), PLB, phosphodiesterase and RyRs. Therefore, NOS and NO^•^ are important regulators of cardiac contractility (106). Deng and Dietrich (2007) (97) showed the effect of NO^•^ production by iNOS on cardiac contractility. Ethanol was shown to bind and inhibit iNOS activity which then augmented cardiac contractility. iNOS also plays an important role in age-related ethanol-induced cardiac dysregulation (106). Acute ethanol administration induced negative inotropic effects in young mouse hearts but positive inotropic effects in the hearts of senescent mice. Senescent mice exhibited increased iNOS activity, hence ethanol -mediated inhibition of iNOS activity is more pronounced in old mice and may offer a cardioprotective effect (106).

Another pathway involved in acute consumption of heavy to moderate concentrations of alcohol is the c-Jun NH (2)-terminal kinase (JNK2) signaling pathway, which is normally activated in response to cellular stress (107). High concentrations of ethanol were shown to increase susceptibility to AF. Ethanol-induced upregulation of JNK2 activity in human and rabbit hearts resulted in amplified phosphorylation of CaMKII. Under physiological conditions, activated CaMKII phosphorylates PLB, causing its dissociation from SERCA, thereby facilitating Ca^2+^-leak from the SR (62). Upregulation of CaMKII activity, therefore, increases SERCA-mediated Ca^2+^-leak, ultimately leading to abnormal Ca^2+^ waves and disruption of E-C coupling (107). Moreover, CYP-2E1 inhibition also revealed the involvement of the JNK and ASK-1 pathways as mediators of the negative inotropic events induced by chronic alcohol administration to mice (88).

#### The Effect of Ethanol Consumption on Ion Channels and Eventual Ca^2+^ Handling

Direct exposure of human atrial muscle strips and mouse ventricular myocytes to ethanol, whether acute or chronic, resulted in the development of the typical hallmarks of negative inotropy, such as perturbed Ca^2+^ homeostasis (56, 87, 90, 107). Human atrial cardiomyocytes acutely exposed to 1–6% ethanol displayed severe Ca^2+^ leak from SR stores, decreased amplitudes of Ca^2+^-transients, decreased myofilament Ca^2+^-sensitivity, and increased NCX activity and SERCA-mediated Ca^2+^ reuptake into the SR. Altogether, these changes led to aberrant E-C coupling and negative inotropism by alcohol (56). In addition, the PI3K/Akt pathway was shown to exert some influence over the oxidative stress induced by acute alcohol consumption (108). It was also postulated that ethanol disturbed intracellular Ca^2+^ homeostasis by favoring RyR2-mediated Ca^2+^-leak, thus reducing the amount of Ca^2+^ to be released from the SR upon stimulation (56). Additionally, the NOX2 pathway and CAMKII activity were shown to be involved in the ethanol-induced upregulation of ROS production in the heart (56, 103, 109, 110). Previous findings from our lab indicated that RyR2 had an ethanol-sensing region and thus intoxicating concentrations of ethanol (18–100 mM) inhibited RyR2 activity (65). The contribution of this ethanol action to alcohol-induced depression of cardiac contractility, however, remains to be determined.

Lastly, it is important to underscore that the morphological and contractile effects of acute ethanol administration on human-induced pluripotent stem cell-derived cardiomyocytes (Hi-PSC-CMs) mimicked many of the effects seem in animal models (111).

Depressed cardiac contractility is also observed after chronic ethanol consumption (87, 88, 93, 104, 112). In FVB mice for example, chronic administration of 4% alcohol caused the classic hallmarks of negative inotropism. However, SERCA levels were decreased (104), in contrast to the upregulated SERCA activity observed after acute ethanol administration (56). Moreover, the protein levels of CYP-2E1, iNOS and PLB increased while NCX levels were downregulated (88). However, these changes in protein levels, along with the aberrant Ca^2+^-handling were significantly ablated in FVB mice expressing an IGF-1 transgene (104). Furthermore, CYP-2E1 and the JNK2 and ASK-1 signaling pathways were implicated in the ethanol-induced upregulation of ROS production after chronic ethanol exposure (88).

The data discussed so far clearly underscores the negative effects of acute and chronic alcohol consumption on cardiac muscle contraction. However, there seems to be a transition period between these two stages of AUD when considering cardiac muscle function (113). Using rat ventricular myocytes, the cardiac effects of ethanol exposure were monitored for different intervals (acute exposure = 10–20 min vs. chronic exposure = 1 and 3 months). Results revealed that the negative inotropic effects of chronic ethanol exposure were biphasic between 1- and 3-months. Both acute and chronic alcohol, however, caused the development of events in ventricular myocytes leading to negative inotropy, i.e., decrease in Ca^2+^ transient amplitude, Ca^2+^ rate of rise and decay of Ca^2+^transients. However, at the 1-month timepoint, indicators of positive inotropy such as increased cell shortening and the augmentation of Ca^2+^ amplitude were observed. In turn, authors concluded that ethanol-mediated decrease in SR load was the main determinant of the sustained negative inotropy in response to chronic alcohol consumption (113).

#### Acetaldehyde and Cardiac Dysfunction

Acetaldehyde is the first product of ethanol metabolism (10, 87, 94). Like ethanol, acetaldehyde impairs E-C coupling in cardiac muscle *via* two main mechanisms. Firstly, acetaldehyde binds proteins to form “protein adducts” which are unstable, nonfunctional and immunogenic, and are therefore degraded *via* the ubiquitin proteasome pathway or autophagy (10, 87, 94). It has been noted that persons suffering from cardiac disorders produce antibodies against these acetaldehyde-associated protein adducts, as well as functional proteins (10, 87, 94). Secondly, acetaldehyde is known to induce negative inotropic events in the heart and is considered to be more potent than ethanol itself, owing to its enhanced bioreactivity with other compounds (91, 100-103). Increased acetaldehyde production disrupts the delicate balance between oxidants and antioxidants. For example, acetaldehyde can be further metabolized to form ROS through the activity of superoxide dismutase, aldehyde oxidase or xanthine oxidase to produce the highly unstable O_2_^−^ (99).

Like ethanol, acetaldehyde also attenuates cardiac contractility (7, 87). For example, O_2_^−^ reacts with NO^•^ to form the highly reactive ROS peroxynitrite which has been shown to damage contractile proteins and many enzymes critical to mitochondrial function (94, 97). Various animal studies have shown that the overexpression and knockdown of ALDH_2_ served to attenuate and augment the effects of both acetaldehyde and O_2_^−^ respectively. Acetaldehyde also modulates PLB protein levels (93) and intracellular Ca^2+^ handling (16,87,101).

#### Influence of Sex on Alcohol-Induced Cardiac Myopathy

Men and women exhibit differences in both ethanol metabolism and the pathogenesis of cardiac myopathies (10, 11, 114). Compared to men, women are more vulnerable to the toxic effects of ethanol abuse (10, 11). Subsequent to acute exposure, Duan et al., (2003) (10) observed that female mice were more sensitive to acetaldehyde-induced hypo-contractility than males. This increased sensitivity is believed to be closely related to estrogen (10, 115). Though there is limited data on the cross reactivity of estrogen and acetaldehyde-induced cardiac activity, estrogen is known to modulate the metabolism of acetaldehyde and increases the production of NO^•^. Cardiac function is heavily modulated by NO^•^ and the release of this ROS augments both ethanol and acetaldehyde-induced cardiac hypercontractility (10).

In the epithelial cells of vascular muscle, estrogen increases NO^•^ production which then augments ethanol-induced effects, therefore this may be the case for cardiac muscle. Females may produce more NO^•^, and may therefore be more prone than males to the deleterious effects of alcohol consumption. These conclusions indicate that both ethanol and its metabolite acetaldehyde attenuate cardiac contractility and these effects can be more potent in women than men (114).

There are many diverse pathways and components involved in the development of cardiomyopathies as a result of ethanol consumption. While the exact mechanisms involved are still unknown, there are many intriguing lines of research that can be explored.

### Alcohol and Vascular Smooth Muscle

While popular knowledge identifies alcohol as a vasodilator, detailed examination of the scientific literature and data from our laboratory challenge this proposition. Indeed, alcohol has been reported to evoke both smooth muscle (SM) relaxation/vasodilation and SM contraction/vasoconstriction depending upon species, vessel type and whether vessels under examination were intact or endothelium-denuded. Additionally, the responses of vessels to alcohol can be attributed to ethanol itself and its vasoactive metabolites, such as acetaldehyde. Indeed, the role of acetaldehyde as a peripheral vasodilator (i.e., skin vessels) and thus mediator of the so-called “alcohol flush” or “oriental flush” in individuals who carry the ALDH2*2 allele, is well established (116-118). Moreover, dilation of arteries by toxicologically relevant concentrations of ethanol has been reported in several vessels, including rat spleen (119) and ewe uterine arteries (120), fetal baboon middle cerebral arteries (MCA) (121), and rat portal veins (122).

In turn, the evidence that toxicologically relevant levels of alcohol induce SM contraction and thus vasoconstriction is overwhelming. *In vitro* findings have demonstrated that alcohol constricts rat cerebral arteries (21, 23, 123, 124), mouse MCA (123-125), rat intracerebral arterioles (126), aorta (127) and coronary arteries (21), dog (128) and pig coronary arteries (129) and human umbilical artery (21). Likewise, abundant *in vivo* data have documented the ability of toxicologically relevant alcohol concentrations to evoke vasoconstriction across different vessel types and species. These include: rat (27, 98), sheep (130) and human cerebral arteries (128), rat skin arteries (119) and human placental vessels (131).

In contrast to the current state of affairs of the literature dealing with skeletal and cardiac muscles, to our knowledge there is no data from studies testing for any possible sex-induced difference in alcohol-induced modulation of SM contractility.

#### Aorta and Coronary Arteries

In the aorta, as in several other vessels (see above), ethanol has been reported to evoke SM contraction and relaxation and thus, vasoconstriction and vasodilation respectively, depending upon species and experimental conditions. Rat aorta strips are constricted by ethanol through a PKC- and calmodulin-dependent mechanism (127). Likewise, acute ethanol administration (1–800 mM) leads to contraction of aortic SM cells, in this case *via* production and release of ROS (O_2_^−^ and H_2_O_2_) from the vessel walls. ROS release in turn, increased the intracellular Ca^2+^concentration *via* a mechanism that is independent of endothelium and involves the cyclooxygenase (COX) pathway (98).

However, the presence of the endothelium cannot solely explain the differential effects of ethanol on aortic diameter and SM tone. For example, in rat aortic rings pre-contracted with either KCl or phenylephrine, Ru et al., (2008) (132) showed that acute administration of 0.1–7% ethanol evoked dilation in both intact and endothelium-denuded thoracic aorta rings; with this effect being more potent in the latter (132). These authors also reported that 2-APB and dantrolene (inhibitors of IP_3_R and RyR respectively), both caused a significant decrease in ethanol-induced aortic dilation, underscoring a possible link between the release of Ca^2+^ from SR stores and ethanol-induced SM relaxation in the aorta. In contrast, Tirapelli et. al. (2006) (133), showed that phenylephrine-induced contraction was augmented in rat aortic rings isolated after chronic administration of 20% (v/v) ethanol. In turn, ethanol levels reflective of mild drinking (2–25 mM) were shown to evoke dilation of both intact and denuded rat aortic rings. This alcohol effect was mediated by ROS-dependent activation of NO^•^ as a consequence of eNOS upregulation by ethanol (24). This study also showed that rat aortic SM cells (VSMCs) could themselves produce NO^•^, which in turn caused vasodilation *via* the cGMP pathway. Furthermore, catalase (a H_2_O_2_ scavenger), Tiron (an O_2_^−^ scavenger), and L-NAME (a nonselective NOS inhibitor) attenuated this ethanol-induced aortic relaxation (24). Likewise, withdrawal from chronic ethanol exposure depressed the contractility of endothelium-denuded aortic rings from rats (134). This reduction in SM tone was independent of O_2_^−^ and H_2_O_2_ signaling, yet it was suggested that the COX-2 pathway could be involved, in a mechanism that was not endothelium-dependent (24).

It is possible to advance that the signaling molecules that participate in ethanol-induced dilation of the aorta, may act upon ion channels which regulate VSMC membrane excitability and thus SM tone. Some voltage-dependent K^+^ channels and ATP-sensitive K^+^ channels are indeed involved in ethanol-induced aortic dilation (54, 135). Regarding SM BK channels, critical determinants of vascular myogenic tone and diameter (54), acute exposure to ethanol (10–100 mM) of native (136) or recombinant bslo1 isoform (137) channels from bovine aortic SM reconstituted into planar lipid bilayers led to a powerful decrease in channel activity, an ethanol action that would lead to increased SM tone and aortic constriction. However, the contribution of ethanol inhibition of BK channel activity to aortic SM tone and diameter is yet to be determined.

With regards to coronary arteries, K_V_ channels have been demonstrated to regulate arterial tone and their activity is modulated by the MAPK signaling pathway (27, 135). Ethanol has been shown to upregulate MAPK activity and decrease K_V_ channel currents, leading to vasoconstriction (27). In addition, inhibition of the MAPK pathway ameliorated ethanol-induced vasoconstriction, thereby positioning the MAPK signaling pathway as a possible therapeutic target in AUD (27).

#### Mesenteric Arteries

Both ethanol and acetaldehyde have been shown to evoke dilation of intact superior mesenteric arteries (SMA) (28,134,138). Acetaldehyde-mediated vasodilation, however, was more potent than that of ethanol (28). A central role for the endothelium in ethanol-induced dilation of mesenteric arteries is underscored by data from Yuui et al. (2019) (138) who showed that chronic administration of moderate levels of ethanol to rats enhanced the activity of an endothelium-dependent hyperpolarizing factor (EDHF) pathway, thereby promoting vascular relaxation. In addition, Jin et al. (2019) (28) showed that ethanol dilation of intact SMA may be mediated through the activity of NO^•^ and guanylyl cyclase, the latter being a main target of EDHF. However, relaxation of SMA in response to ethanol was also evoked in de-endothelialized vessels (129, 132).

In contrast to the findings described in the previous paragraph, pre-treatment of intact mesenteric resistance arteries from mice with either ethanol or acetaldehyde leads to increased efficacy of the vasopressor phenylephrine (i.e., favoring mesenteric artery constriction) (139). The molecular mechanisms underlying this ethanol action remain to be established.

#### Carotid Arteries

Chronic ethanol consumption has been shown to impair carotid artery relaxation *via* upregulation of the potent vasoconstrictor endothelin-1 (ET-1) (133). Of note, ET-1 is involved in pro-inflammatory and mitogenic processes, and its dysregulation has been implicated in several disorders of the cardiovascular system (140, 141). The study by Tirapelli et al. (2006) (133) showed that chronic administration of 20% (v/v) ethanol led to enhanced ET-1 production in endothelium-intact rat carotid rings, which in turn increased carotid artery constriction. Additionally, phenylephrine-induced contraction was not enhanced further in the presence of ethanol. The exact mechanism by which ethanol influences ET-1 activity is unknown. However, while neither the pre- nor post-transcription production of ET-1 was unchanged, the protein levels of the ETB receptor which regulates dilation in carotid arteries was significantly decreased (133).

#### Cerebral Arteries

As described and referenced in the beginning of this section, constriction of cerebral arteries in response to acute exposure to toxicologically relevant concentrations of ethanol (10–100 mM) is widespread, and is observed across different species and vessel types (e.g., cortical vessels, parenchymal arterioles, etc.). This drug action is largely mediated by ethanol itself rather than its vasoactive metabolites. It is important to underscore that heavy alcohol consumption has been linked to the induction of brain hypoperfusion in humans (142), systemic arterial hypertension (the main risk factor for stroke), and cerebral events including cerebral infarction and/or hemorrhage (23).

As outlined previously, Ca^2+^ plays a critical role in the regulation of smooth muscle contraction and vascular tone, including that of cerebral arteries (25, 123, 125, 143). Thus, it is not surprising that most studies pursuing a mechanism(s) to explain alcohol-induced cerebrovascular constriction have focused on ion channels and signaling molecules that control Ca^2+^ homeostasis in these vessels. For example, Yang et al. (2001) (143) observed that ethanol-induced constriction of canine basilar arteries was modulated by both SR Ca^2+^-release (*via* InsP_3_ or RyR) and extracellular Ca^2+^ influx *via* voltage-gated Ca^2+^-channels. Ca^2+^ release from SR stores was transient while extracellular Ca^2+^ influx into the cytoplasm was prolonged, with both events mediating basilar artery constriction. While activation of voltage-gated Ca^2+^-channels would lead to cerebral artery constriction, there is no such evidence from available literature. In fact, our laboratory demonstrated that ethanol at concentrations that constricted MCA (50 mM) failed to modify voltage-gated Ca^2+^-channel activity in MCA SM (23).

In cerebral artery SM, however, Ca^2+^/voltage-gated K^+^ of big conductance (BK) channels play a key role in controlling Ca^2+^ homeostasis, myogenic tone and cerebral artery response to vasomodulators (23, 52, 54, 61). BK channels are activated by membrane depolarization and/or local vasodilatory Ca^2+^ signals (termed “sparks”) that are released from activated SR RyR channels. Eventually, activated BK channels generate spontaneous transient outward currents (STOCS) which lead to repolarization of the membrane, inactivation of VDCCs and inhibition of external Ca^2+^ influx. The cumulative results of these events are the blunting of SM contraction while relaxation and vasodilation are favored (52, 54, 144).

Using freshly isolated myocytes from rat MCA and isolated vessel segments, our laboratory demonstrated that inhibition of MCA STOCS and eventual MCA constriction by intoxicating levels of ethanol (50 mM) did not involve alcohol metabolites and was independent of the endothelium. Rather, this effect was due to ethanol-induced inhibition of both RyR-generated sparks (23, 65) and β_1_ subunit (encoded by *KCNMB1*)-containing BK channels (23, 125). Indeed, *KCNMB1*−/− mice exhibited a significant reduction in ethanol-induced inhibition of BK-induced STOCs and its resulting vasoconstriction (125). These results identified the BK β_1_ subunit as a possible therapeutic target to counteract alcohol-induced inhibition of SM BK channels and its associated cerebrovascular constriction, a proof-of-principle being obtained both *in vitro* and *in vivo* data with celastrol, a neuroprotective agent (145).

Remarkably, IP_3_-mediated Ca^2+^ waves were not affected by toxicologically relevant levels of ethanol (23), therefore underscoring the selectivity of alcohol actions towards SR RyR and BK channels. In follow-up studies (65), we also documented that toxicologically relevant ethanol concentrations reduced the steady-state activity of recombinant RyR2 (the isotype that prevails in MCA SM (146). Equivalently, this effect was also shown using an RyR2 truncation mutant consisting only of the channel functional core and its activation domain (75). These results suggest that RyR2s present a delimited region that senses the presence of ethanol.

Alcohol, however, interacts with many endogenous compounds to exert its effects on cerebral arteries. Thus, some endogenous molecules may actually protect against the ethanol-induced increase in SM contraction and its associated cerebral artery constriction. Cholesterol (CLR), for example, alleviated ethanol-mediated contraction of myocytes isolated from MCA and eventual vessel constriction in mice through a mechanism that was not dependent on BK β_1_ subunits (25). This CLR-mediated protection was further confirmed using mice that were maintained on a high fat diet; when both statins and 50 mM ethanol were co-administered, CLR levels were pointedly decreased in excised MCAs. This CLR-mediated vascular effect was accompanied by significantly increased arterial constriction (123, 147), and shown to be associated with PKC signaling pathway(s).

However, the enhancement of SM tone and cerebrovascular constriction by alcohol, is not limited to RyR, BK channels and their interconnecting signaling. The transient receptor potential cation channel subfamily V member 1 (TRPV1) is widely expressed in arterial blood vessels and is another ion channel that modulates ethanol-induced cerebrovascular activity (124). Ethanol and the stimulant caffeine are usually consumed together and it has been demonstrated that TRPV1 participates in the actions of both caffeine and ethanol in MCA. Their co-administration revealed the protective effect of caffeine against ethanol-induced MCA constriction, which was suggested to occur *via* a mechanism involving NO^•^-mediated activation of TRPV1 (124, 148). Taken together, our findings underscore the central role of TRPV1 in the vasoactive properties of two of the most widely consumed recreational drugs in the world: caffeine and alcohol.

#### Uterine Arteries

Pre-and peri-natal alcohol consumption increases the risk of impaired fetal cognitive functions and the development of Fetal alcohol syndrome (FAS) (149). However, a critical part of pregnancy is the remodeling of the uterine SM arteries, which facilitate the delivery of nutrients and gases to the developing fetus. In a healthy mother, this remodeling is characterized by the enlargement of the CSA of uterine arteries and a decrease in the media:lumen ratio (26, 150, 151). Chronic ethanol consumption restricts this uterine remodeling and increases myogenic constriction *via* unknown mechanisms. These developments are indicative of artery remodeling (26, 150, 151) and increase the risk of fetal undernutrition, miscarriage, low birth weight and the development of FAS (26). Furthermore, impaired ROS signaling is a mediator of ethanol-induced dysregulation of muscle contractility, and increased iNOS and NO^•^ production have been implicated in the development of FAS (97).

### Alcohol and Non-Vascular Smooth Muscle

The effects of alcohol on non-vascular smooth muscle are diverse and differ among tissue types or even within the same tissue. These differences are primarily observed according to the duration of ethanol exposure (acute or chronic) and the concentration of ethanol used. Below, we outline the prevailing wisdom on the ethanol-induced effects on non-vascular smooth muscle contractility, and the known mechanisms by which this is facilitated.

#### Gastrointestinal Tract

Alcohol abuse has many deleterious effects on the entire gastrointestinal tract. Some of these outcomes include; duodenal hemorrhage, and damage to the mucosal lining thus causing impaired gut permeability and increased endotoxin levels (152). In turn, these processes upregulate inflammatory mediators such as macrophages, resulting in a proinflammatory milieu. Acute alcohol consumption is associated with improper absorption of glucose, amino acids, deficiencies in vitamins such as B12, B6, vitamins C, A, D, E and K, and an overload of intracellular iron stores. In addition to these effects, chronic alcohol consumption causes malabsorption of macronutrients (carbohydrates, proteins and lipids), as well as increased absorption of H_2_O and Na^+^ in the small intestine resulting in diarrhea.

The interplay between ethanol and different reactive nitrogen intermediates has been of particular interest to researchers (153, 154). The vast majority (~95%) of the ethanol that reaches the liver is oxidized, thereby allowing its elimination form the body (9, 12, 13, 153). Ethanol metabolism also occurs in the stomach, where O_2_^−^ and NO^•^ interact to produce the potent oxidizing agent peroxynitrite. The nitrosation of ethanol by peroxynitrite, produces the potent vasodilator ethyl nitrite (153, 154). Using gastric fundus strips, Gago et al., (2008) (154) showed that administration of nitrite (NO_2_^−^), the precursor of NO^•^, caused minor vasodilation. In contrast, acute ethanol exposure resulted in minor vasoconstriction. However, co-administration of the two compounds resulted in a significant increase in vasodilation owing to the formation of ethyl nitrite. The authors therefore, concluded that their findings demonstrated the protective effect of wines and brandy in the gut, when consumed in moderation (154). For further discussion of the vascular effects of ethanol on the GI or other body systems, please see the section on vascular smooth muscle.

It has also been demonstrated that ethanol causes both relaxation and constriction in a dose-dependent manner (155-157). Low concentrations of ethanol (25–100 mM) decreased smooth muscle contractility in both human, cat and canine esophageal muscle (155). This hypo-contractility was believed to be caused by the inhibition of Ca^2+^ influxes.

Conversely, data obtained in guinea pigs, showed that a higher ethanol concentration (342 mM) caused gastric smooth muscle constriction (156). The high ethanol concentration was reflective of the levels found in the gut due to the direct diffusion of ethanol across the mucosal surface, as compared to the lower ethanol levels found in blood. The authors advanced that this ethanol-induced constriction occurred through a phospholipase A2 (PLA_2_)-mediated mechanism, since PLA_2_ inhibitors decreased this effect. However, the specifics of this mechanism are still unknown. Likewise, 20–500 mM ethanol caused constriction of both longitudinal and circular smooth muscles of guinea pigs (156). This action was shown to be dependent on extracellular Ca^2+^ and believed to be mediated *via* a tyrosine kinase signaling pathway.

#### Bladder

There is limited information about the mechanism(s) by which alcohol dysregulates smooth muscle contractility in the bladder since the relationship between alcohol and bladder cancer is the primary focus of research (158). Impaired detrusor contractility (IDC) is a condition which manifests as intermittent voiding (emptying of the bladder), increased bladder capacity, decreased volumes of released urine and increased residual volume (22, 159). Acute administration of ethanol to male rats caused impaired detrusor contractility and mimicked many of the effects observed in IDC (22). Likewise, alcohol has also been shown to decrease detrusor muscle contractility in response to different ligands (22, 160). However, chronic ethanol exposure of detrusor muscle strips from rat bladder caused muscle constriction, which was facilitated by the flux of Ca^2+^ from both intracellular stores and extracellular medium (160). These studies point to dysregulation of E-C coupling, resulting in impaired smooth muscle contractility, but the particular mechanisms involved remain unknown.

#### Lung

Chronic alcohol consumption increases the risk of developing pneumonia, lung infections and injury. In animal studies, chronic exposure to ethanol *via* inhalation caused cellular dysfunction and oxidative stress (161). Additionally, the phagocytic activity of alveolar macrophages was severely impaired leading to increased susceptibility and intensity of pneumonia. In addition, ethanol was also found to promote apoptosis in the epithelial cells of parenchymal tissue (161).

Regarding contractile events, ethanol has been shown to induce dilation within lung smooth muscle (162, 163). In lung parenchymal smooth muscle, chronic alcohol administration (*via* inhalation), was reported to decrease contractile force (163). The Rho-associated protein kinase (ROCK) pathway mediates “Ca^2+^ sensitization”, which is the increase in contractile force by signaling pathways that are independent of supplementary increases in intracellular Ca^2+^ (164). Ethanol attenuated this ROCK-mediated Ca^2+^ sensitization mechanism, which led to reduced contraction in lung parenchymal smooth muscle. Finally, in cultured rat airway smooth muscle cells, acute exposure of 100 mM ethanol caused relaxation *via* a cGMP/PKG mechanism (162).

#### Vas Deferens

The link between alcohol consumption and erectile dysfunction (ED) has been studied for years (165-170). The corpus cavernosum (CC) of the vas deferens are cavernous spaces which fill with blood to facilitate tumescence. In addition to the constriction of penile arteries, this increase in intracorporeal pressure is also dependent on the relaxation of non-vascular smooth muscle cells (169, 170). The chronic exposure of rats to ethanol (5–20% in diet) caused morphological changes in the smooth muscle myocytes, such as a decreased number of elastic fibers and collagen type 4 (170), and decreased smooth muscle area along with increased expression of Caspase 3 therefore increased apoptosis (169). As a result, these factors affected CC smooth muscle contractility and have been implicated in alcohol-induced ED.

Another protein which modulates the contraction of CC trabecular smooth muscle cells is endothelin1 (ET-1) along with other members of the endothelin pathway. ET-1 binds to ET_A_ and ET_B_ receptors which regulate contraction and relaxation respectively. Increased levels of these pathway constituents is associated with the development of ED. ET-1 causes vasoconstriction by binding to ET_A_ which activates NAD(P)H, resulting in the production O_2_^−^ which promotes vasoconstriction. O_2_^−^ is very unstable so is quickly converted by superoxide dismutase (SOD) and catalase to H_2_O_2_ which has vasodilation properties (171).

Chronic ethanol consumption has been shown to increase ET-1 expression and upregulate ET_A_ activity while also decreasing SOD activity and increases CAT activity (166, 168, 171). The cumulative result of this ethanol-induced effect is increased O_2_^−^ production, which promotes vasoconstriction and decreased production of the vasodilatory H_2_O_2._ leading to increased CC contraction (166). Cyclooxygenase (COX) pathway mediators such as prostanoids also play a pivotal role in mediating dilation in trabecular smooth muscle cells and promote vasodilation through the endothelin pathway. Chronic ethanol consumption impairs COX pathway activity resulting in decreased prostanoid production which alleviates the vasodilatory influence on the endothelin pathway leading to CC contraction. There is also some evidence that ET-1 -mediated vasoconstriction may involve the ROCK pathway (168).

With respect to ethanol-induced modulation of E-C coupling, it was shown that acute and heavy alcohol consumption in peri-adolescent male Wistar rats caused decreased Ca^2+^ signaling in the vas deferens (172). A similar ethanol-induced impairment of E-C coupling was reported for the prostate and epididymis (167, 172). This impaired ethanol-induced Ca^2+^ influx was found to be mediated primarily by DHPR with little involvement of IP_3_R- and RyR-controlled SR Ca^2+^ stores (167).

## SUMMARY AND DISCUSSION

In this review, we aimed to present the most recent and widely understood viewpoints on the mechanisms involved in alcohol-induced disturbance of skeletal, cardiac and smooth muscle contractility. The consumption of alcohol is both enjoyed and abused globally, yet both acute and chronic consumption of moderate-large quantities of alcohol are solidly proven to have deleterious effects on the contractility of all three muscle types. A common feature of contractile dysfunction among all muscle types is the disruption of Ca^2+^ homeostasis and EC-coupling. In contrast, there still seems to be disparate opinions on the beneficial effects that low quantities of alcohol drinking might exert on heart contractility and their underlying mechanisms.

In striated muscle, the general consensus is that ethanol and/or acetaldehyde attenuate myocyte contractility, leading to muscle weakness and, in the long term, muscular atrophy. In smooth muscle however, the narrative is much more complex: in vascular smooth muscle cells, ethanol generally exerts both vasodilatory (uterine, mesenteric, etc.) and vasoconstrictive (coronary, cerebral, carotid, and uterine arteries) effects. However, in nonvascular smooth muscle, ethanol-mediated vasodilation is observed in the gut and lung while vasoconstriction is seen in the vas deferens. To further complicate matters, ethanol-induced SM contraction and vasoconstriction vs. SM relaxation and vasodilation involve many of the same biochemical signaling molecules, such as ROS, production of nitrogenous compounds (NO^•^, peroxynitrite, ethylnitrite), ethanol detoxification enzymes (CYP-2E1, ADH, ALDH_2_, catalase), and changes in the activity of ion channels (RyRs, DHPRs, SERCA, PLB, NCX etc), proteostasis, mitochondrial function and increased autophagy. Additionally, among the many studies presented, the different methodologies used, the time course of ethanol administration (acute vs. chronic) and other experimental variables, makes it problematic to compare, analyze and therefore reach conclusive viewpoints.

Another concept that is common among researchers, is that the harmful effects of acute ethanol consumption are reversible while chronic consumption leads to permanent damage. There are of course caveats to these rules as revealed in the cases of HHS and the French paradox. Moreover, the difference in alcohol metabolism between men and women, predisposes the latter to more severe manifestations of ethanol-induced myopathies and is a recognized phenomenon. However, these observed differences may be a direct result of ethanol’s association with different downstream mediators/ pathways. For example, in striated and smooth muscle, both ethanol and acetaldehyde exert control over the activities of various signaling pathways, resulting in the modification of muscle contractility. These include the endothelin, JNK_2_, NOX2, iNOS, EDGF, RhoA/Rho-kinase, PLA_2_, estrogen and c-Myc pathways among others.

From the various studies conducted, some trends are apparent: the RhoA/Rho-kinase pathway has been implicated in ethanol-induced effects in smooth muscle myocytes in the lung, carotid artery and vas deferens, leading to SM contraction. Likewise, in striated muscle, the influence of ethanol and acetaldehyde on increased ROS and NO^•^ production, seems to be a common factor that then causes aberrant Ca^2+^ homoeostasis, EC-coupling and proteostasis, resulting in attenuated myocyte contractility and loss of muscle mass.

In conclusion, alcohol exerts potent and complex effects on skeletal, cardiac and smooth muscle myocyte contractility, which are facilitated by a myriad of biological players and downstream signaling pathways. A lot of research lies ahead in order to suggest a rationale for therapeutic interventions to counteract muscle contractility impairment in AUD.
